# Type 5 Familial Hemophagocytic Lymphohistiocytosis in a Seven-year-old Girl Post Second Bone Marrow Transplantation with Failure to Thrive: STXBP2 Novel Mutation

**DOI:** 10.7759/cureus.6246

**Published:** 2019-11-27

**Authors:** Abdullah Baothman, Hani Almalki, Khalid Abumelha, Abobaker Alshegifi, Abdulrahman Baashar

**Affiliations:** 1 Pediatrics, King Abdullah International Medical Research Center, Jeddah, SAU; 2 Internal Medicine, King Abdullah International Medical Research Center, Jeddah, SAU; 3 Internal Medicine, King Abdulaziz Medical City, Jeddah, SAU; 4 Internal Medicine: Diabetes and Endocrinology, King Saud bin Abdulaziz University for Health Sciences, Makkah, SAU

**Keywords:** hemophagocytic lymphohistiocytosis, stxbp2, chronic diarrhea

## Abstract

Familial hemophagocytic lymphohistiocytosis (HLH) is a fatal autosomal recessive disorder resulting in an exaggerated and ineffective immune response. Genetic defects in familial HLH can lead to the impaired function of the secretory lysosome-dependent exocytosis pathway. We report an STXBP2 homozygous missense mutation c.1139A>G, p.(Gln380Arg) consistent with a genetic diagnosis of familial hemophagocytic lymphohistiocytosis type 5 associated with chronic diarrhea in a seven-year-old girl. She was diagnosed with HLH and achieved remission by the HLH-2004 protocol and allogeneic matched bone marrow transplantation (BMT) from her sibling. However, six years later, she had a relapse of HLH, which required a second BMT. Ever since then, she continued to have persistent chronic watery diarrhea and failure to thrive. Patients with familial HLH type 5 due to STXBP2 gene mutation can manifest as either with or without chronic diarrhea. This unusual relationship directs toward a specific gene mutation of STXBP2 as the cause of chronic diarrhea in familial HLH. The prevalence of familial HLH in Saudi Arabia is underestimated. Due to the high rate of consanguinity and the local customs of marrying within the same community, clinicians should consider familial HLH as a cause of persistent, unexplained, chronic diarrhea among the pediatric age group.

## Introduction

Hemophagocytic lymphohistiocytosis (HLH) is a rare and fatal immune disorder that is characterized by a hyperinflammatory immune response associated with the unregulated and sustained activation of lymphocytes and macrophages. In 1939, Scott and Robsmith described four adult cases of HLH, which were thought to be an atypical Hodgkin’s disease, namely, histiocytic medullary reticulosis [1]. Thirteen years later, Farquhar and Claireaux described two pediatric cases of histiocytic medullary reticulosis in infancy [2]. Since then, HLH was categorized into the primary, including familial and primary immune deficiency with HLH, and secondary forms [3]. Familial HLH is an autosomal recessive condition with incomplete penetrance. Genetic mutations have been associated with five subtypes of familial HLH, resulting in the impaired function of the secretory lysosome-dependent exocytosis pathway, including the mutation of chromosome 9 in type 1, PFR1 in type 2, UNC13D in type 3, STX11 in type 4, and STXBP2 in type 5 [4]. Other primary immunodeficiency syndromes are linked to HLH, including Chediak-Higashi syndrome, Griscelli syndrome type 2, Hermansky-Pudlak syndrome type 2, X-linked lymphoproliferative disease types 1 and 2, and Wiskott-Aldrich syndrome [5]. Secondary HLH is associated with infections, malignancies, and rheumatologic causes [5]. The majority of familial HLH cases were diagnosed during infancy, with an incidence of 1.1:100,000 in patients younger than one year. The median age of onset in those patients is six months [6]. In patients of Southern European descent (70%) and North Americans, the PRF1, UNC13D, and STXBP2 mutations are the most common [7-8]. To our knowledge, there are no prevalence studies about familial HLH in Saudi Arabia. However, two cohort studies in Saudi Arabia reported a higher incidence of familial than secondary HLH due to high consanguinity rates and positive family history with the identification of new novel mutations [9-10]. We report a rare novel mutation of STXBP2 in a seven-year-old girl following her second bone marrow transplant, associated with chronic diarrhea.

## Case presentation

A seven-year-old girl, who is a product of a term pregnancy and a daughter of a consanguineous marriage, developed metabolic acidosis, diarrhea, and pancytopenia in the postnatal period. She was diagnosed with HLH and achieved remission by the HLH-2004 protocol and allogeneic matched bone marrow transplantation (BMT) from her sibling at two months of age. However, six years later, she suffered from relapse due to rejection, which required a second BMT. Family history revealed two healthy siblings, and three deceased: two sisters, aged two months and six years, and a seven-month-old brother, all due to HLH; all were associated with chronic diarrhea.

After the second BMT, she was brought to the emergency department complaining of frequent loose bowel motions, persistent vomiting, and rhinorrhea for five days. Physical examination showed generalized weakness, rhinorrhea, dry mucous membranes, and the appearance of a malnourished child but vitally stable, with no fever.

Her initial blood work revealed normal white blood cell (WBC) count, red blood cell (RBC) count, hematocrit, and hemoglobin. However, there was thrombocytopenia, increased ferritin level, increased C-reactive protein level, decreased total protein, and low albumin (Table 1).

**Table 1:** Initial investigation at the time of presentation AST: aspartate aminotransferase; ALT: alanine aminotransferase

Initial investigation	Patient’s Reading	Reference Range
Platelet	76 × 109/L	150 – 450 × 109/L
Ferritin	605 ug/L	4-120 ug/L
C-reactive protein	46 mg/L	0.0 – 5.0 mg/L
Potassium	2.2 mmol/L	3.1 – 5.1 mmol/L
Sodium	134 mmol/L	135-144 mmol/L
Chloride	119 mmol/L	101 – 111 mmol/L
Calcium	1.88 mmol/L	2.20 – 2.70 mmol/L
Phosphate	1.09 mmol/L	1.32 – 1.91 mmol/L
Magnesium	0.63 mmol/L	0.70 – 0.86 mmol/L
Carbon dioxide	7 mmol/L	20 – 28 mmol/L
Alkaline phosphate	507 U/L	156 – 369 U/L
AST	22 IU/L	18 – 36 IU/L
ALT	45 U/L	9 – 25 U/L
Total protein level	46 g/L	60 – 80 g/L
Albumin	27 g/L	38 – 54 g/L
Serum creatinine	45 μmol/L	27 – 62 μmol/L
Blood urea nitrogen	1.2 mmol/L	2.5 – 6.0 mmol/L

She was admitted as a case of dehydration, with electrolyte disturbance and acidosis. An intravenous (IV) bolus of 200 mL of normal saline was given, followed by IV fluids and 10 meq of potassium chloride. Eventually, the patient required total parenteral nutrition (TPN) through a central venous line for persistent electrolyte disturbance. As for the cause of chronic diarrhea, the patient underwent several investigations in the hope of reaching a specific diagnosis. The cultures of blood and stool were all negative. Rotavirus antigen and Clostridium difficile toxins were also negative. Stool microscopy reported no white blood cells, red blood cells, and no presence of ova or parasites, as well as a negative culture of Helicobacter pylori. A sigmoidoscopy and upper endoscopy with multiple biopsies were performed to rule out graft-versus-host disease (GVHD) and viral infections. The results of these biopsies showed no features supportive of a GVHD diagnosis. The results of polymerase chain reaction (PCR) for cytomegalovirus (CMV), Epstein-Barr virus (EBV), and herpes simplex virus (HSV) were also negative.

Due to the clinical emergency and chronicity of diarrhea, serology for TB-Quantiferon was done, as the patient was going to receive a trial of infliximab. However, it was stopped after five doses, as there was no improvement in her symptoms. As a result, TPN therapy was initiated daily, except for Friday and Saturday. To minimize hospital visits, a shift-to-home TPN was planned after educating the family.

**Figure 1:**
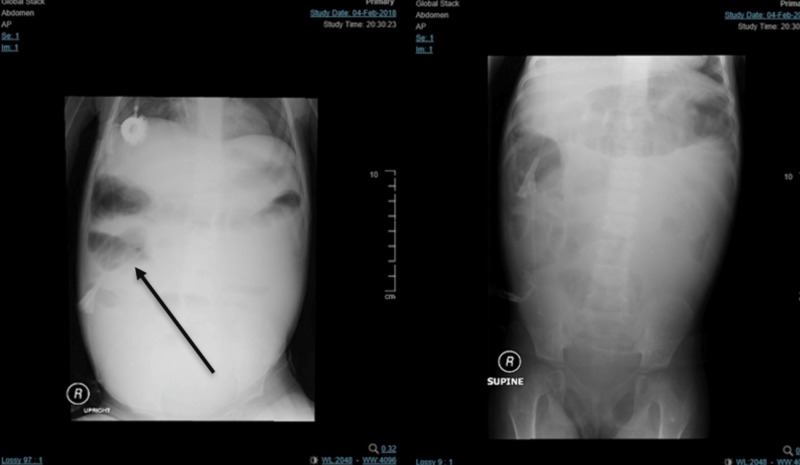
Erect (left) and supine (right) abdominal X-ray showing multiple air-fluid levels scattered all over the abdomen with increased mid abdominal opacifications.

A buccal swab for whole-exome sequencing (WES) analysis showed STXBP2 homozygous missense mutations c.1139A>G, p.(Gln380Arg) consistent with a genetic diagnosis of familial hemophagocytic lymphohistiocytosis type 5. The patient was diagnosed with small bowel bacterial overgrowth evidenced by the radiological manifestation of bowel obstruction and a high level of B12 of 648 pmol/L (reference range, 131 - 507 pmol/L) (Figure 1).

A multidisciplinary team was conducted and recommended managing this case with an intestinal transplant or a permanent TPN on the ward, as she had a clinical picture of intestinal failure secondary to HLH-type 5. Unfortunately, the family refused both treatment options, and the patient was started on TPN for five days every other week. The patient improved on intermittent TPN. This approach has reduced her episodes of diarrhea and electrolyte imbalance. However, the patient is still falling below her growth chart with poor weight gain and short stature; height: 103 cm and weight: 13.9 kg at the age of eight years, both parameters below the third percentile. She was prescribed a trial of growth hormone due to delayed bone age.

## Discussion

Familial hemophagocytic lymphohistiocytosis (HLH) is a fatal autosomal recessive disorder resulting in an exaggerated and ineffective immune response. Genetic defects in familial HLH can lead to the impaired function of the secretory lysosome-dependent exocytosis pathway [4]. The clinical features of HLH are defined in HLH-2004 criteria (Table 2) [11]. The diagnosis of HLH can be made by either molecular studies or by the fulfillment of five out of eight clinical features of HLH-2004 diagnostic criteria. The implementation of the molecular study and the identification of the genetic mutation of familial HLH is important since not all patients fulfill the clinical diagnostic criteria but still respond to the HLH-2004 treatment protocol.

**Table 2:** Histiocyte Society HLH-2004 Diagnostic Criteria HLH: hemophagocytic lymphohistiocytosis

The patient fulfilled five out of the following critera:		
Molecular diagnosis of genetic mutations consistent with HLH		
Splenomegaly		
Fever		38.5 C or more
Cytopenia (affecting at least two cell lineages):	Hemoglobin	< 90 g/L (in infants < 4 weeks; Hg < 100 g/L)
	Platelets	< 100 x 10^9/L
	Neutrophils	< 1.0 x 10^9/L
Hypertriglycemia and/or hypofibrinogenemia:	Fasting triglycerides	> 3.0 mmol/L
	Fibrinogen	< 1.5 g/L
Hemophagocytosis in bone marrow, spleen, lymph nodes, or liver		
Low or absent natural killer cells activity		
Ferritin		> 500 mg/L
Soluble CD25 (i.e. IL-2)		> 2400 U/mL

Our patient had a molecular diagnosis of HLH via WES. In addition to that, she had cytopenia affecting two cell lineages, hemoglobin and platelets, high ferritin, and low fibrinogen.

Consanguineous marriage is a significant risk factor for autosomal recessive diseases. Familial HLH was observed in 75% and 77% in two retrospective cohort studies in Saudi Arabia [9-10]. Ali Al Ahmari et al. identified a pattern of the recurrent identification of the STX11, STXBP2, and UNC13D mutations based on the population’s tribal and geographical distribution. Furthermore, STXBP2 mutations were identified in the majority of Saudi patients, with 10 other novel mutations in the same study. Nevertheless, larger-scale multicenter studies should be conducted to estimate the prevalence of familial HLH and associated mutations.

Mutations in the STXBP2 gene, which encodes syntaxin-binding protein (Munc18-2), have been linked to the development of familial HLH type 5 [12]. This protein plays a major role in the regulation of intracellular granule trafficking in epithelial cells, neutrophils, and mast cells [13]. The indexed patient’s mutation STXBP2 variant c.1139A>G p.(GIn380Arg) is classified as a variant of uncertain significance (class 3) according to the recommendations of the Centogene and American College of Medical Genetics (ACMG) (Table 3). Patients with familial HLH type 5 demonstrate variable hematological and gastrointestinal symptoms [14]. Chronic diarrhea has been reported in patients with familial HLH type 5, in addition to other atypical features, including sensorineural hearing loss and abnormal bleeding [15-17]. Defects in Munc18 can affect the platelets' function and secretion, which explains the persistent thrombocytopenia observed in the indexed case [18]. There are different homozygous and compound heterozygous mutations that have attributed to an abnormal STXBP2 gene encoding. Pagel et al. conducted a cohort study of 37 patients with familial HLH type 5 and discovered that 14 patients had gastrointestinal symptoms in the form of chronic diarrhea that resulted in a failure to thrive in the first months of life. Diarrhea persisted in six out of eight patients despite treatment with hematopoietic stem cell transplant (HSCT). Similarly, our patient continued to have persistent chronic watery diarrhea despite undergoing a second bone marrow transplant. Interestingly, patients with familial HLH type 5 due to STXBP2 gene mutation have an all-or-none phenomenon in regard to intestinal symptoms. In families with more than one affected child, intestinal symptoms were present either in all or none of the affected offspring. This unusual relationship points toward mutations of STXBP2 as the cause of chronic diarrhea in familial HLH type 5 [19]. All siblings in the indexed case in whom the diagnosis of HLH was made had chronic diarrhea and intestinal symptoms. Unfortunately, the patient’s siblings did not have a molecular diagnosis of their HLH, as the diagnosis was made clinically based on HLH-2004 diagnostic criteria. Various mutations reported for patients with familial HLH type 5 associated with and without chronic diarrhea are summarized in Table 4 [20]. These data are reported in an open-access registry available at (www.mvid-central.org).

**Table 3:** Detailed description of the index patient mutations of STXBP2

Gene	Variant Coordinates	In-Sluice Parameters	Allele Frequencies	Type and Classification
STXSP2	Chr19(GRCh37):g.7708130A>G	PolyPhien: Probably damaging	gnomAD: -	Missense
	NM_001272034.1:c.1139A>G	Align-GVGD: C35	ESP -	Uncertain significance
	p.(Gin380Arg)	SFT: Deleterious	1000 G -	(Class 3)
		MutationTaster Disease-causing	CentoMD -	
		Conservation: nt moderate/aa high		
		2/3 possible splice effect		

**Table 4:** Mutations of STXBP2 in familial HLH type 5 associated with and without chronic diarrhea

STXBP2 with Chronic Diarrhea			STXBP2 Without Chronic Diarrhea		
Mutation	Effect	Consequence	Mutation	Effect	Consequence
c.474del10bpinsGA	p.Cys237Wfs*76	Frameshift	c.87+2T>C	NA	Splicing
c.326-24del8bp	NA	Frameshift	c.1621G>A	p.Gly514Ser	Missense
c.1099-1107del9bp	p.Val367-Gln369del	Deletion	c.310A>T	p.Ile104Phe	Missense
c.901+6T>G	NA	Splice site	c.1621G>A	p.Gly541Ser	Missense
c.1214G>A	p.Arg405Gln	Missense	c.875G>A	p.Arg292His	Missense
c.693-695del3bp	p.Ile232del	Deletiond	c.1430C>T	p.Pro477Leu	Missense
c.279delG	p.Thr94Profs*25	Frameshift	c.260delT	p.Leu87Argfs*32	Frameshift
c.502dupC	p.Gln168Profs*71	Frameshift	c.1247-1G>C	p.Val417Leufs*126	Frameshift
c.1430C>T	p.Pro477Leu	Missense	c.626T>C	p.Leu209Pro	
c.1696+1G>A	NA	Splice site	c.1247-1G>C	p.Val417LeufsX126	Frameshift
c.1601T>C	p.Leu534Pro	Missense	c.706delG	p.Ala236GInfs*24	Frameshift
c.1621G>A	p.Gly541Ser	Missense	c.1247-1G>C	p.Val417Leufs*126	Frameshift
c.1727delT	p.Phe576Serfs*5	Frameshift	c.1247-1G>C	p.Val417Leufs*126	Missense
c.1213C>T	p.Arg405Trp	Missense	c.1621G>A	p.Gly541Ser	Missense
c.1247-1G>C	p.Val417Leufs*126	Frameshift	c.1247-1G>C	p.Val417Leufs*126	Frameshift
c.1213C>T	p.Arg405Trp	Missense	c.1247-1G>C	p.Val417Leufs*126	Frameshift
c.1247-1G>C	p.Val417Leufs*126	Frameshift	c.1724_1729del GC TTCC	p.Arg575_Phe576del	Deletion
c.37+1G>A	NA	Splice site			
c.902+5G>A	NA	Splice site			
c.1146delC	p.Lys383Argfs*4	Splice site			
c.1139A>G ^(^^§)^	p.(Gln380Arg)	Missense			
(§): The reported mutation in the index patient Reference: Dhekne HS, Pylypenko O, Overeem AW, et al. MYO5B, STX3, and STXBP2 mutations reveal a common disease mechanism that unifies a subset of congenital diarrheal disorders: A mutation update. Hum Mutat. 2018;39(3):333‐344.					

Despite the improvement in the overall survival rate after the introduction of the HLH-2004 protocol, 30-day mortality remained high. Both elevated Th1 and Th2 and interleukin-10 (IL-10) were identified as a marker of increased risk of death in HLH [20]. Furthermore, other independent prognostic risk factors of early mortality included hypoalbuminemia (≤27.75 g/L), elevated lactate dehydrogenase (LDH) (≥3707.5 U/L), and increased IL-10 (≥456 pg/mL) at diagnosis. In the presented case, the girl was suffering from hypoalbuminemia (30 g/L) and elevated LDH levels (1161 U/L). After the second BMT, her albumin levels initially went further down (to 18 g/L) and remained persistently low even though there was a slight increase in the next reading (34 g/L). On the other hand, the LDH levels got dramatically low after the second BMT (183 U/L), and it remained within the normal range in the next readings (188 U/L).

Familial HLH is commonly treated using the HLH-2004 protocol, which consists of an eight-week induction phase followed by a continuation phase. Both phases consisted of etoposide, cyclosporine, intrathecal methotrexate, and corticosteroids. This intensive regimen aims to provide favorable conditions for hematopoietic stem cell transplantation. Our patient received dexamethasone daily, starting with 10 mg/m^2^ for the first two weeks, then 5 mg/m^2^ for the following two weeks, then 2.5 mg/m^2^ for the following two weeks, 1.25 mg/m^2^ for one week, and tapering during week eight. Additionally, she received a pulse steroid every other week with 10 mg/m^2^ for three days. She also received cyclosporin A, aiming at a level of around 200 mcg/L, starting with 6 mg/kg daily divided into two doses. In addition to the previous treatment, she monthly received IV immunoglobulin of 0.5 g/kg. The chronic diarrhea was initially managed with TPN every other week, and then it became once weekly every month.

## Conclusions

Although familial HLH is a rare disorder, its prevalence in Saudi Arabia is still undetermined. Due to the high rate of consanguinity and the local customs of marrying within the same community, clinicians should consider familial HLH as a cause of persistent, chronic diarrhea among the pediatric age group. Patients with familial HLH type 5 who are suffering from chronic diarrhea can be managed with intermittent TPN to improve the patient's quality of life and decrease the admission rate. However, further studies are required to validate this approach.

## References

[1] Scott RB, Robb-Smith AHT (1939). Histiocytic medullary reticulosis. Lancet.

[2] Farquhar JW, Claireaux AE (1952). Familial haemophagocytic reticulosis. Arch Dis Child.

[3] Janka G, Lehmberg K (2014). Hemophagocytic syndromes--an update. Blood Rev.

[4] Allen CE, McClain KL (2014). fl-ILH: becoming a blended family. Blood.

[5] Mehta RS, Smith RE (2013). Hemophagocytic lymphohistiocytosis: a review of literature. Med Oncol.

[6] Erker C, Parker-Murray P, Talano JA (2017). Usual and unusual manifestations of familial hemophagocytic lymphohistiocytosis and Langerhans cell histiocytosis. Pediatr Clin North Am.

[7] Jordan MB, Allen CE, Weitzman S, Filipovich AH, McClain KL (2011). How I treat hemophagocytic lymphohistiocytosis. Blood.

[8] Sieni E, Cetica V, Hackmann Y (2014). Familial hemophagocytic lymphohistiocytosis: when rare diseases shed light on immune system functioning. Front Immunol.

[9] Al Ahmari A, Alsmadi O, Sheereen A (2017). Novel molecular changes in Saudi patients with familial hemophagocytic lymphohistiocytosis. J Blood Disord Transfus.

[10] Elyamany G, Alzahrani A, Elfaraidi H, Alsuhaibani O, Othman N, Al Mussaed E, Alabbas F (2016). Hemophagocytic lymphohistiocytosis: single-center series of 12 cases from Saudi Arabia. Clin Med Insights Pediatr.

[11] Henter JI, Horne AC, Arico M (2007). HLH-2004: diagnostic and therapeutic guidelines for hemophagocytic lymphohistiocytosis. Pediatr Blood Cancer.

[12] zur Stadt U, Rohr J, Seifert W (2009). Familial hemophagocytic lymphohistiocytosis type 5 (FHL-5) is caused by mutations in Munc18-2 and impaired binding to syntaxin 11. Am J Hum Genet.

[13] Cote M, Menager MM, Burgess A (2009). Munc18-2 deficiency causes familial hemophagocytic lymphohistiocytosis type 5 and impairs cytotoxic granule exocytosis in patient NK cells. J Clin Invest.

[14] Vogel G, van Rijn J, Krainer I (2017). Disrupted apical exocytosis of cargo vesicles causes enteropathy in FHL5 patients with Munc18-2 mutations. JCI Insight.

[15] Pagel J, Beutel K, Lehmberg K (2012). Distinct mutations in STXBP2 are associated with variable clinical presentations in patients with familial hemophagocytic lymphohistiocytosis type 5 (FHL5). Blood.

[16] Bezdjian A, Bruijnzeel H, Pagel J, Daniel S, Thomeer H (2018). Low-frequency sensorineural hearing loss in familial hemophagocytic lymphohistiocytosis type 5. Ann Otol Rhinol Laryngol.

[17] Al Hawas R, Ren Q, Ye S, Karim Z, Filipovich A, Whiteheart S (2012). Munc18b/STXBP2 is required for platelet secretion. Blood.

[18] Sandrock K, Nakamura L, Vraetz T, Beutel K, Ehl S, Zieger B (2010). Platelet secretion defect in patients with familial hemophagocytic lymphohistiocytosis type 5 (FHL-5). Blood.

[19] Dhekne HS, Pylypenko O, Overeem AW (2018). MYO5B, STX3, and STXBP2 mutations reveal a common disease mechanism that unifies a subset of congenital diarrheal disorders: a mutation update. Hum Mutat.

[20] Tang Y, Xu X, Song Song (2008). Early diagnostic and prognostic significance of a specific Th1/Th2 cytokine pattern in children with haemophagocytic syndrome. Br J Haematol.

